# NtMYB4 and NtCHS1 Are Critical Factors in the Regulation of Flavonoid Biosynthesis and Are Involved in Salinity Responsiveness

**DOI:** 10.3389/fpls.2019.00178

**Published:** 2019-02-21

**Authors:** Shuai Chen, Fengyan Wu, Yiting Li, Yanli Qian, Xuhao Pan, Fengxia Li, Yuanying Wang, Zhenying Wu, Chunxiang Fu, Hao Lin, Aiguo Yang

**Affiliations:** ^1^Tobacco Research Institute, Chinese Academy of Agricultural Sciences, Qingdao, China; ^2^Qingdao Institute of Bioenergy and Bioprocess Technology, Chinese Academy of Sciences, Qingdao, China; ^3^Biotechnology Research Institute, Chinese Academy of Agricultural Sciences, Beijing, China

**Keywords:** NtMYB4, *NtCHS1*, flavonoid pathway, ROS level, salt stress

## Abstract

High levels of salinity induce serious oxidative damage in plants. Flavonoids, as antioxidants, have important roles in reactive oxygen species (ROS) scavenging. In the present study, the tobacco R2R3 MYB type repressor, NtMYB4, was isolated and characterized. The expression of *NtMYB4* was suppressed by salinity. Overexpression of *NtMYB4* reduced the salt tolerance in transgenic tobacco plants. NtMYB4 repressed the promoter activity of *NtCHS1* and negatively regulated its expression. Rutin accumulation was significantly decreased in *NtMYB4* overexpressing transgenic plants and *NtCHS1* RNAi silenced transgenic plants. Moreover, high H_2_O_2_ and ${\text{O}}_{2}^{-}$ contents were detected in both types of rutin-reduced transgenic plants under high salt stress. In addition, exogenous rutin supplementation effectively scavenged ROS (H_2_O_2_ and ${\text{O}}_{2}^{-}$) and improved the salt tolerance of the rutin-reduced transgenic plants. In contrast, *NtCHS1* overexpressing plants had increased rutin accumulation, lower H_2_O_2_ and ${\text{O}}_{2}^{-}$ contents, and higher tolerance to salinity. These results suggested that tobacco NtMYB4 acts as a salinity response repressor and negatively regulates *NtCHS1* expression, which results in the reduced flavonoid accumulation and weakened ROS-scavenging ability under salt stress.

## Introduction

Salinity is one of the most significant abiotic stresses affecting plant growth and agricultural productivity. The response of plants to salinity is a complex set of traits that involves signaling and metabolic processes at molecular, cellular, and whole-plant levels. Extensive research has been carried out on the mechanisms of plant salt tolerance, including ion homeostasis, osmotic adjustment, reactive oxygen species (ROS) scavenging, detoxification, signal transduction, transcription, and crosstalk with other stresses (Tuteja, 2007; Zhang et al., 2012; Deinlein et al., 2014). However, the detailed mechanism underlying plant salt tolerance is unclear.

The exposure of plants to salinity results in massive changes in gene expression. Transcription factors (TFs) are initially vital in sensing salt and stimulating tolerance responses (Deinlein et al., 2014). TFs, including MYB, WRKY, bHLH, bZIP, and NAC families, are differentially expressed in response to salinity (Jiang and Deyholos, 2009; Jiang et al., 2009; Yang et al., 2009; He et al., 2012; Cui et al., 2013; Deinlein et al., 2014). These TFs regulate the expression levels of various genes that may function in salt tolerance of plants. Studies have demonstrated that gene manipulation could alter the salt tolerance of plants. For example, the ectopic expression of TaMYB73 from wheat in *Arabidopsis thaliana* activates the expression of stress signaling genes such as *AtCBF3* and *AtABF3*, and enhances the tolerance to ionic and salinity stress (He et al., 2012). In turn, the TF AtbZIP24, which down-regulates the expression of stress inducible-genes such as the Na^+^ transporter *AtHKT1;1*, is required during salt stress in plants. In this trend, AtbZIP24 silenced transgenic *Arabidopsis* shows increased salt tolerance compared to wild type (WT) plants (Yang et al., 2009). In the same line, the ethylene responsive factor BpERF11 down-regulates the expression of important genes involved in abiotic stress tolerance such as *LEA* and *DHN*, and leads to reduced levels of proline and the accumulation of ROS. Thus, BpERF11 negatively regulates plant salt and osmotic tolerance (Zhang et al., 2016).

Flavonoids are important secondary metabolites in plants and have important roles in the resistance to oxidative damage caused by ROS during plant growth and abiotic stresses due to their antioxidant activity (Moore et al., 2005; Yu and Jez, 2008; Agati et al., 2012; Pandey et al., 2015). It has been reported that flavonols modulate ROS levels to control the stomata aperture (Watkins et al., 2017) and the formation of root hairs (Maloney et al., 2014). Accumulation of flavonoids, such as flavonols, anthocyanins, and proanthocyanidins in plants could enhance salt tolerance (Liu et al., 2014; Li P. et al., 2017). In this trend, moderate salt stress was shown to induce the accumulation of flavonoids and promote the quality of agricultural crops and medicinal plants (Lim et al., 2012; Colla et al., 2013). Rutin is a glycoside flavonoid that is abundant in a wide variety of plants. Rutin has received much attention due to its health benefits, such as activity against diabetes and inflammation, prevention of hypertension, and antioxidant properties (Kerdudo et al., 2014; Yoo et al., 2014; Kaur and Muthuraman, 2016; Ghorbani, 2017). Rutin biosynthesis is regulated by flavonoid structural genes and TFs. Six structural genes, *PAL*, *C4H*, *4CL*, *CHS*, *CHI*, and *FLS*, are the major genes involved in the rutin biosynthetic pathway (Winkel-Shirley, 2001). Rutin is one of the most abundant flavonoids, accounting for more than 90% of total flavonoids in tobacco (Li et al., 2014). For this reason, tobacco is the proper model plant to explore the function of rutin in salt tolerance.

Increasing evidence has indicated that R2R3 MYB TFs play important roles in regulating the flavonoid biosynthetic pathway in plants (Dubos et al., 2010; Zhang et al., 2014; Zhou et al., 2017a). R2R3 MYB subgroup 4 TFs, AtMYB3, AtMYB4, AtMYB7, and AtMYB32 are involved in repression of the phenylpropanoid and flavonoid pathways in *Arabidopsis* (Jin et al., 2000; Dubos et al., 2010; Fornalé et al., 2014; Zhou et al., 2017b). Recently, many genes orthologous to *AtMYB4*, including *ZmMYB31*, *ZmMYB42*, *EgMYB1*, *BrMYB4*, *CsMYB4a*, and *FtMYB11*, were identified from different plants, all of which negatively regulate phenylpropanoid biosynthesis (Fornalé et al., 2006; Legay et al., 2010; Zhang et al., 2014; Li M. et al., 2017; Zhou et al., 2017a). To the best of our knowledge, tobacco MYB repressors have not been studied yet.

In the present study, we identified an R2R3 MYB TF, NtMYB4, which is orthologous to AtMYB4. We determined that the expression of the *NtMYB4* gene was significantly repressed by high salinity. In addition overexpression of *NtMYB4* resulted in reduced rutin accumulation, which affects the antioxidant ability of plants under salt stress. Our results indicate that the reduced rutin contents might be a consequence of the repression of the biosynthetic gene *NtCHS1* by NtMYB4. As a whole, these data indicate that NtMYB4 plays an important function during the responses of plant to saltine stress.

## Materials and Methods

### *NtMYB4* Gene Isolation

A search for sequences orthologous to AtMYB4 was conducted using the Blastp tool in China Tobacco Genome Database^1^. The isolated MYB protein sequences were aligned and MYB proteins containing the conserved LLsrGIDPxT/SHRxI/L, EAR repression, zinc-finger (CX_1–2_CX_7–12_CX_2_C), and GY/FDFLGL motifs in the C-termini were selected for further analysis (Jin et al., 2000; Zhou et al., 2015). PCR was used to clone the full length cDNA of NtMYB4 with the primers NtMYB4-F/NtMYB4-R (Supplementary Table S1). The PCR products were purified and cloned with a pEASY-Blunt Cloning Kit (TransGen Biotech, China) and then sequenced. RNA isolation from tobacco leaves, cDNA synthesis, and PCR were performed as described in Chen et al. (2017). Protein sequences were aligned in DNAMAN software (Lynnon Biosoft, United States). A phylogenetic tree was constructed with the neighbor-joining (NJ) algorithm in the MEGA6.0 program (Kumar et al., 2008). The robustness of the tree topology was assessed using 1,000 bootstrap replicates.

### Genetic Construction and Plant Transformation

For the preparation of cassettes for the overexpression of *NtMYB4* and *NtCHS1* and for the silencing of *NtCHS1*, each gene-specific amplicon was amplified from tobacco leaf cDNA, synthetized as reported in the following paragraph. In order to construct the overexpression vector *NtMYB4*-pCHF1, the entire coding sequence (CDS) of *NtMYB4* was amplified using primers NtMYB4OE-F/NtMYB4OE-R (Supplementary Table S1). The PCR product was digested with X*ba*I/*SacI* and cloned into the X*ba*I/*SacI* digested pCHF1 plasmid under the control of the CaMV 35S promoter. To construct the overexpression vector *NtCHS1*-pC3301-ZDS, coding region (CDS) of *NtCHS1* (Chen et al., 2017) was amplified using primers NtCHS1OE-F/NtCHS1OE-R (Supplementary Table S1). The PCR product was digested with X*ba*I/*SacI* and cloned into the X*ba*I/*SacI* digested pC3301-ZDS plasmid under the control of the CaMV 35S promoter. A 293 bp long fragment of *NtCHS1* was amplified using the gene-specific primers NtCHS1RNAi-F/NtCHS1RNAi-R, which contain attB sites (underlined) (Supplementary Table S1). Using BP and LR reactions, the target fragment was then ligated into the destination vector pH7GWIWG2(I) (Xu et al., 2011) according to the Gateway^TM^ manufacturer’s instructions (Invitrogen, Carlsbad, CA, United States), to yield the *NtCHS1-RNAi* vector. The pCHF1-*NtMYB4*, pC3301-ZDS-*NtCHS1* and NtCHS1-RNAi vectors were introduced into *Agrobacterium tumefaciens* strain LBA4404. Transformation of tobacco was performed using the leaf disc method as reported by Horsch et al. (1985).

In order to construct the subcellular localization vector *NtMYB4*-CPB-YFP, NtMYB4-YFP-F/NtMYB4-YFP-R primers (Supplementary Table S1) were used to amplify the NtMYB4 coding region and the PCR product was cloned into the BamHI digested pCPB-YFP plasmid using the ClonExpress^®^ Entry One Step Cloning Kit (Vazyme Biotech, China).

With the purpose of constructing the reporters and effectors for UAS/GAL4-based transcriptional repression assay. NtMYB4BD-F/NtMYB4BD-R primers (Supplementary Table S1) were used to amplify the NtMYB4 coding region. For constructing the pGBKT7-*NtMYB4* vector, the PCR product was cloned into the BamHI digested pGBKT7 plasmid using the ClonExpress^®^ Entry One Step Cloning Kit (Vazyme Biotech, China). To construct the effector vectors, two primer pairs GAL4BD-F/GAL4BD-R and GAL4BD-F/PMDC32-NtMYB4-R (Supplementary Table S1) were used to amplify the fusion sequence from pGBKT7-*NtMYB4*. Then the fusion sequences were cloned into the BamHI digested pMDC32 plasmid using the ClonExpress^®^ Entry One Step Cloning Kit. The reporters 35S-UAS::GUS were constructed according to the method of Chen et al. (2014).

With the purpose of constructing the reporters and effectors for the dual luciferase assay, seven primer pairs pPAL-F/pPAL-R, pC4H-F/pC4H-R, p4CL-F/p4CL-R, pCHS1-F/pCHS1-R, pCHI-F/pCHI-R, pFLS-F/pFLS-R, and pANS-F/pANS-R (Supplementary Table S1) were designed to amplify the promoter sequences of the genes *NtPAL*, *NtC4H*, *Nt4CL*, *NtCHS1*, *NtCHI*, *NtFLS*, and *NtANS*, respectively. For pGreen vectors construction, the promoter sequences of seven genes were cloned into the BamHI digested pGreen plasmid using the ClonExpress^®^ Entry One Step Cloning Kit.

### Quantitative RT-PCR Analysis (qRT-PCR)

Total RNA was extracted from WT tobacco tissue, such as leaves, roots, stems, buds and flowers, and from transgenic tobacco leaves according to the method described in Chen et al. (2017). First-strand cDNA was synthesized using a PrimeScript^TM^ RT Master Mix (Perfect Real Time) (Takara, Clontech, Japan) according to the manufacturer’s instructions. The qRT-PCR was performed using SYBR^®^ Premix Ex Taq^TM^ (Tli RNaseH Plus) (Takara, Clontech, Japan), and primers listed in Supplementary Table S1. The *Tob103* gene (GenBank accession no. U60495) served as an internal control. The reaction mixtures consisted in 10 μl SYBR Green mix, 0.4 μl forward qRT-PCR primer, 0.4 μl reverse primer, 1 μl template cDNA, and 7.8 μl sterile water. The thermal cycling parameters were 95°C for 30 s, followed by 40 cycles of 95°C for 15 s and 58°C for 34 s. The relative expression levels were normalized to the expression of the *Tob103* gene. The comparative cycle threshold (ΔΔCT) method was used to calculate the relative expression levels of the target genes. Data were expressed as the mean ± SD as determined from three independent biological replicates.

### Subcellular Localization

NtMYB4-CPB-YFP construct was transformed into *A. tumefaciens* EHA105 strain. A single bacterial clone was grown at 28°C overnight, centrifuged for 10 min at 4,200 rpm, re-suspended in 10 mM MgCl_2_/10 mM MES to a final OD = 1.0 and cultured for 2–3 h with 2 μl 100 mM acetosyringone in the dark. Transformed bacteria were infiltrated into leaves of *Nicotiana benthamiana* (Chen et al., 2014). The leaves were collected between 48 and 72 h after infiltration and examined by confocal laser scanning microscopy. Images of triplicate infiltrated leaves were acquired with a Fluo View^TM^ FV1000 microscope equipped with an argon laser line of 515 nm (excitation) for YFP signal.

### Dual Luciferase Assay

The binding to promoters of flavonoid structural genes was assessed to test the transcriptional regulation activity of NtMYB4. Five promoter-pGreen vectors, pCHF1-*NtMYB4*, pCHF1 empty vector, and P19 were transformed into *A. tumefaciens* AH105. The bacteria were grown with 2 μl 100 mM acetosyringone at 28°C overnight. To prepare the suspension used for infiltration, bacteria were re-suspended in 10 mM MgCl_2_/10 mM MES to a final OD = 1.0. Transformed bacteria containing pGreen, pCHF1 and P19 vectors were combined at a 3:3:1 volume ratio, cultured for 1–2 h in the dark and then infiltrated into leaves of *N. benthamiana* as reported above. The leaves were harvested for dual luciferase assay analysis at 72 h after infiltration according to the instructions of the Dual-Luciferase^®^ Reporter Assay System (Promega, United States).

### UAS/GAL4-Based Transcriptional Repression Assay

A transient assay was carried out to test NtMYB4 transcriptional repression activity. To prepare the suspension used for infiltration, bacteria were re-suspended in 10 mM MgCl_2_/10 mM MES to a final OD = 1.0. Transformed bacteria containing 35S-UAS::GUS, GAL4BD/GAL4BD-*NtMYB4* and P19 vectors were combined at a 2:2:1 volume ratio and cultured for 2–3 h in the dark. Transformed bacteria were infiltrated into leaves of *N. benthamiana* and cultured under weak light conditions. The leaves were harvested for GUS staining at 72 h after infiltration (Liu et al., 2010).

### Plant Growth and Salt Treatment

Seeds of WT and transgenic plants of *Nicotiana tabacum* cultivar Honghuadajinyuan (HD) were sterilized in 75% ethanol for 30 s and then washed three times in sterile water. After that, they were further sterilized in 15% (w/v) H_2_O_2_ solution for 8 min and then washed three times in sterile water. For the germination test, the sterilized seeds were cultured on MS solid medium with 0 mM NaCl, 200 mM NaCl, or 200 mM NaCl + 100 μM rutin, respectively. The germination rate was measured in the next 0–11 days after sowing. For the root length test, the sterilized seeds were cultured on 1/2 MS solid medium. Ten day-old seedlings were then transferred to MS solid medium with 0 mM NaCl, 200 mM NaCl, or 200 mM NaCl + 100 μM rutin. The plates were cultured in growth chambers under a 16 h light/8 h dark photoperiod at 28°C. Root length was measured at 20 days after transfer. Forty seeds were used per experiment, and all experiments were repeated three times independently. For the analysis of *NtMYB4* expression under salt stress, seedlings with three or four leaves grown on basal MS medium were transferred to a liquid MS medium containing 200 mM NaCl for 12 or 24 h. The corresponding control was prepared by incubating seedlings in MS liquid medium for the same time. Leaves were harvested after 0, 12, 24 h, and 30 days of treatments according to the method of Chen et al. (2017). All collected samples were frozen in liquid nitrogen and stored at −80°C.

### Measurements of H_2_O_2_ and ${\text{O}}_{2}^{-}$ Content

Collected leaves of 1-month-old plants treated with high salt stress (200 mM NaCl) were used to measure ROS levels. The measurement of H_2_O_2_ content was based on the method described by Libik et al. (2005). The ${\text{O}}_{2}^{-}$ content was determined by measuring the formation of red azo compound, which has specific absorption peak at 530 nm, according to the ${\text{O}}_{2}^{-}$ Detection Kit (SA-1-G, Keming, China). In addition, H_2_O_2_ and ${\text{O}}_{2}^{-}$ were detected visually using the 3’3’-diaminobenzidine (DAB) and nitro blue tetrazolium (NBT) histochemical staining method as described by Zhao et al. (2016). For measurements of each treatment, leaves were collected from at least three plants.

### Measurement of Rutin by High-Performance Liquid Chromatography

Rutin was extracted according to the method described by Li et al. (2015), with some modifications. Dried leaves samples (0.1 g) were extracted with 1 ml of 80% ethanol by 20 min at 40°C with ultrasound, followed by a new extraction with 95% ethanol for 10 min, and then three washes with 80% ethanol 10 min. Supernatants were collected and the volume brought to 50 ml by distilled water and filtered in a 0.45 μm, 13 mm Millex Syringe Filter (Merck Millipore, Carrigtwohill Co., Cork, Ireland). Rutin contents in extracts were analyzed by HPLC as described previously (Huang et al., 2016). Analysis was carried out on three independent biological replicates each containing three technical replicates.

### Measurement of Anthocyanin Content

The flowers were collected in the full-blossom period of WT and *NtCHS1* transgenic tobacco. Anthocyanin from flowers were measured by spectrophotometry according to the method of Pattanaik et al. (2010). All samples were measured as triplicates in three independent biological replicates.

### Statistical Analysis

Data were analyzed by Duncan’s multiple range tests in the ANOVA program of SPSS (IBM SPSS 22). The *P*-value less than 0.05 and 0.001 was considered statistically significant.

## Results

### Characterization of NtMYB4, a Nuclear Localized Transcription Repressor

**FIGURE 1:**
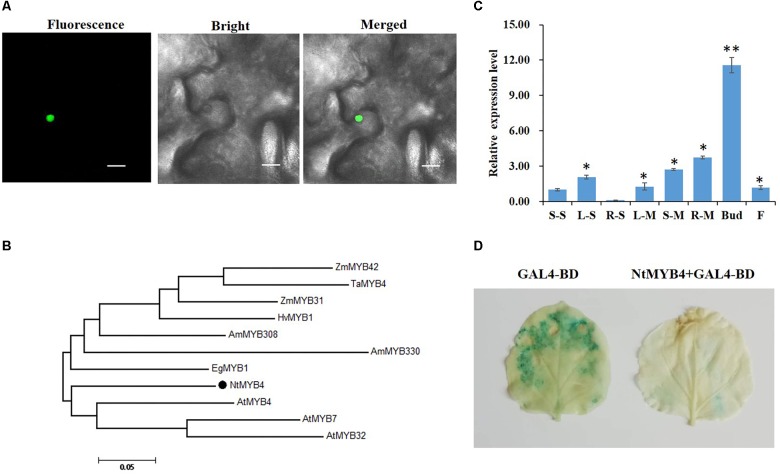
NtMYB4 is a nuclear localized transcription repressor in tobacco. **(A)** Subcellular localization analysis of NtMYB4. An NtMYB4-GFP construct was transformed into leaves of *N. benthamiana* and examined by confocal laser scanning microscopy. A confocal micrograph is shown at the left (green fluorescent protein, GFP), the corresponding differential interference contrast (bright) image is in the middle, and the merged image is at the right. Bars, 20 μm. **(B)** Phylogenetic tree based on the amino acid sequences of NtMYB4 and 10 other R2R3 MYB subgroup 4 members. The NtMYB4 isolated in this study is highlighted by a black dot. Accession numbers for MYB subgroup 4 sequences are: AmMYB308 (JQ0960), AmMYB330 (P81395), AtMYB4 (AY519615), AtMYB7 (AEC06531), AtMYB32 (NP_195225), EgMYB1 (CAE09058), HvMYB1 (P20026), TaMYB4 (AAT37167), ZmMYB31 (CAJ42202), and ZmMYB42 (CAJ42204). **(C)** The expression pattern of NtMYB4 in tobacco root, stem, leaf flower and bud was detected by qRT-PCR. Expression data were detected in two representative growth stages of common tobacco, including the seedling (S) and mature (M) stages. Organs sampled included leaf (L), stem (S), root (R), flower (F). Asterisks in **(C)** indicate differences between tissues (^∗^*P* < 0.05, ^∗∗^*P* < 0.01). **(D)** UAS/GAL4-based transcriptional repression assay of NtMYB4 in *N. benthamiana* leaves. NtMYB4 repressed the 35S-driven GUS activity. Pairwise combinations of constructs as indicated were co-infiltrated into tobacco leaves and stained for GUS activity. The left leaf marked GAL4-BD is 35S-driven GAL4 DNA binding domain (BD) as control; the right leaf marked NtMYB4+GAL4-BD was 35S-driven full-length NtMYB4 fused to the Gal4-BD domain.

By blasting the tobacco genome database with AtMYB4 amino acid sequence, we identified a R2R3 MYB, NtMYB4, as orthologous to AtMYB4. An alignment of amino acid sequences of NtMYB4 and three other orthologous sequences revealed that it contained the R2 and R3 conserved motifs in the N-terminal region, and the conserved LLsrGIDPxT/SHRxI/L motif, EAR repression motif, zinc-finger domain (CX_1–2_CX_7–12_CX_2_C), and GY/FDFLGL motif in the C-terminal region (Supplementary Figure S1). A phylogenetic analysis was performed using the amino acid sequences of NtMYB4 and 10 other orthologous sequences. The result indicated that NtMYB4 clustered with AtMYB4, AtMYB7, and AtMYB32 (Figure 1B), which are members of subgroup 4 and repressors of the phenylpropanoid pathway. Subcellular localization analysis showed that NtMYB4 was a nuclear localized protein (Figure 1A). Spatial transcript accumulation analysis showed that in seedlings *NtMYB4* was more expressed in leaves, whereas in mature plants in all organs assayed, especially the floral bud (Figure 1C). A UAS/GAL4-based transcriptional repression assay showed that NtMYB4 had strong repression activity in tobacco leaves (Figure 1D). These results suggested that NtMYB4 might act as a nuclear localized transcription repressor in tobacco.

### *NtMYB4* Overexpression Significantly Reduced Salt Tolerance

**FIGURE 2:**
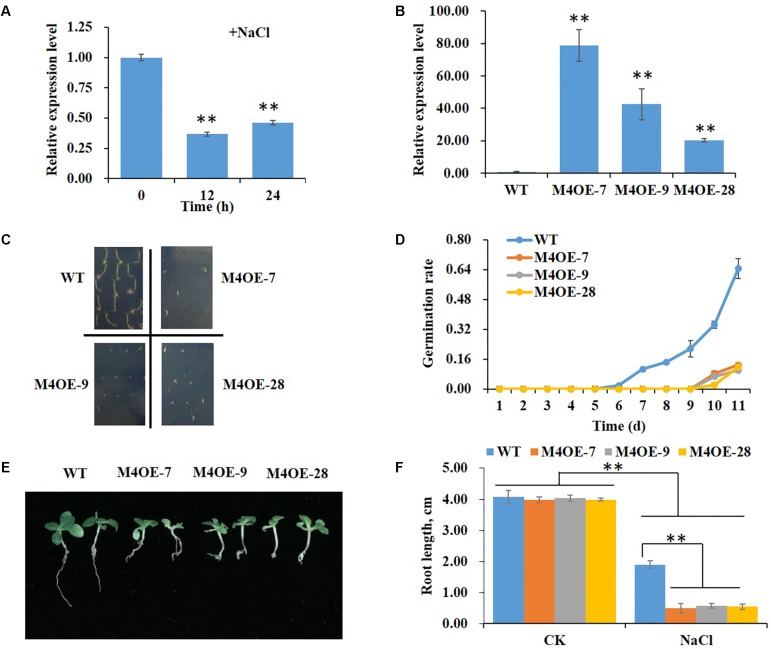
Overexpression of *NtMYB4* significantly reduces salt tolerance. The relative expression levels were normalized to the expression of the *Tob103* gene (internal control). The relative expression of *NtMYB4* in leaves of WT plants was set to 1. **(A)** The expression of *NtMYB4* in leaves of WT plants was significantly suppressed by the addition of 200 mM NaCl in the medium. Asterisks in **(A)** indicate significant difference (^∗∗^*P* < 0.01) between control condition and salt treatment. **(B)** The expression level of *NtMYB4* in leaves was significantly increased in overexpressing transgenic tobacco compared to the WT. **(C,D)** The seed germination of WT and three *NtMYB4* overexpressing transgenic tobacco lines under 200 mM NaCl. **(E,F)** The root elongation of WT and three *NtMYB4* overexpressing transgenic tobacco lines under 200 mM NaCl. Data are expressed as the mean ± SD as determined from three independent biological replicates. Values in **(F)** that are significantly different between treatments and among lines within each treatment are marked with ^∗∗^*P* < 0.01 and ^∗^*P* < 0.05.

Real-time PCR was performed to determine changes in expression levels of *NtMYB4* in WT tobacco in response to high salt stress. The result showed that the level of *NtMYB4* mRNA in leaves was significantly lower after 12 and 24 h of seedling treatment with 200 mM NaCl (Figure 2A).

To verify the function of NtMYB4 in salt tolerance, we overexpressed the *NtMYB4* gene in tobacco. Three transgenic lines M4OE-7, M4OE-9, and M4OE-28 were obtained (Figure 2B) and analyzed in detail. The germination rate and root length of WT and transgenic seeds under high salt stress treatment were measured. Even though there were no obvious differences in germination rates between WT and transgenic seeds when grown on basal MS medium (0 mM NaCl), severe suppression was observed in NtMYB4 overexpressing transgenic seeds under 200 mM NaCl as the germination rate was decreased 92% (Figure 2C). Moreover, we also observed that salt stress provoked a delay in germination, which normally occurs at the fourth day. Thus, WT seeds started to germinate on the sixth day in MS amended with 200 mM NaCl, while the NtMYB4 overexpressing transgenic seeds on the ninth day (Figure 2D). On basal medium, no obvious differences in seedling growth and root length were observed between WT and transgenic plants (Figure 2F). Conversely, 200 mM NaCl treatment caused a significant inhibition in root elongation in both WT and transgenic lines, being 53, 87, 86, and 86% the inhibition observed in WT, M4OE-7, M4OE-9, and M4OE-28, respectively. Interestingly, under salt treatment, the root length was significantly reduced in all transgenic lines with respect to WT plants (Figure 2E,F). These results showed that overexpression of *NtMYB4* reduced the seed germination rate and root elongation under 200 mM NaCl compared to WT, which indicated that NtMYB4 plays a negative role in plant salt tolerance.

### *NtMYB4* Overexpression Leads to Reduced Flavonoid Accumulation

**FIGURE 3:**
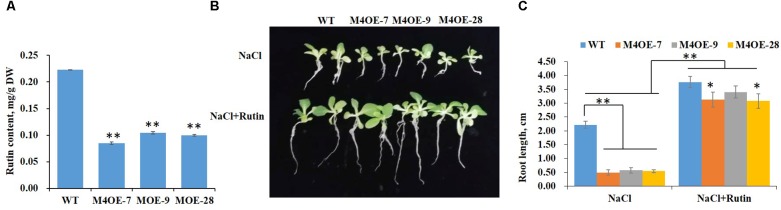
Supplementation with rutin increases salt tolerance of tobacco. **(A)** Rutin accumulation was significantly decreased in the leaves of *NtMYB4* overexpressing transgenic tobacco lines compared to WT. DW, dry weight. Asterisks in **(A)** indicate significant difference (^∗∗^*P* < 0.01) between NtMYB4-OE and WT. **(B,C)** The root length of WT and three *NtMYB4* overexpressing transgenic lines under 200 mM NaCl with or without 100 μM rutin. Data are expressed as the mean ± SD as determined from three independent biological replicates. Values in **(C)** that are significantly different between treatments and among lines within each treatment are marked with ^∗∗^*P* < 0.01 and ^∗^*P* < 0.05.

Numerous reports have demonstrated that salt stress can induce the accumulation of phenolic compounds in plant tissues (Ksouri et al., 2007; Baâtour et al., 2013). The key structural genes of the phenylpropanoid pathway *PAL*, *CHS*, *CHI*, *DFR*, *FLS*, and *ANS* were induced by salt stress in leaves of WT tobacco (Supplementary Figure S2A). Moreover, rutin accumulation increased significantly under 200 mM NaCl in tobacco (Supplementary Figure S2B). In order to further determine the effects of salt stress in flavonoid biosynthesis, we investigated rutin accumulation in NtMYB4 overexpressing transgenic plants. Rutin content significantly decreased in *NtMYB4* overexpressing transgenic tobacco compared to WT (Figure 3A). We speculated that rutin might play a significant role in plant salt tolerance. In order to verify this hypothesis, we supplemented culture media with 100 μmol exogenous rutin. Under these experimental conditions, the inhibition of root length elongation was partially counterbalanced (Figure 3B,C), and plants were more robust overall (Figure 3B). Rutin supplementation in fact significantly reduced the inhibition of root length elongation observed both in WT and transgenic lines on MS with 200 mM NaCl. Only one, M4OE-9, of the three transgenic lines showed root length similar to WT plants on MS supplemented with 200 mM NaCl and 100 μmol rutin (Figure 3C). These result showed that supplementation with rutin enhances the salt tolerance of both WT and *NtMYB4* overexpressing transgenic tobacco seedlings.

### Rutin Effectively Scavenges ROS Under Salt Stress

**FIGURE 4:**
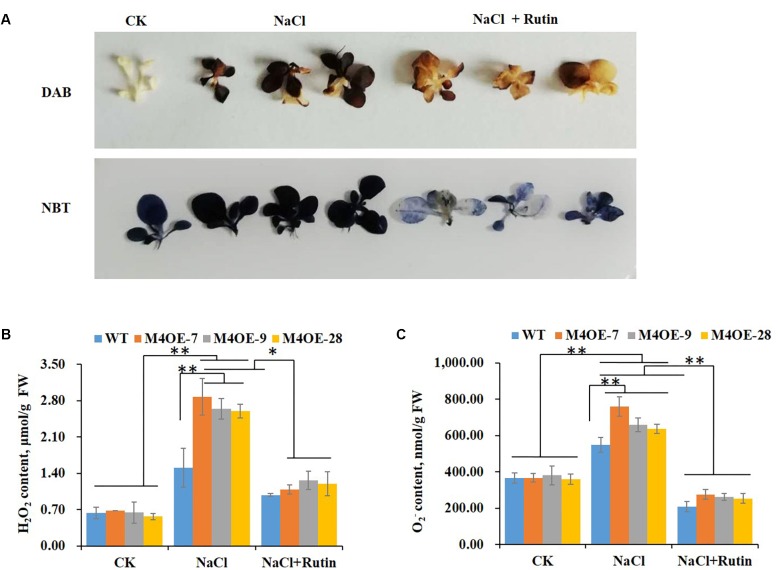
Rutin effectively scavenges the ROS levels under salt stress. **(A)** DAB or NBT staining of WT plants under control (CK) and salt (NaCl) treatment with or without rutin supplementation. **(B,C)** H_2_O_2_ or ${\text{O}}_{2}^{-}$ content of WT plants and *NtMYB4* overexpressing transgenic lines under control (CK) and salt (NaCl) treatment with or without rutin supplementation, measured by visible spectrophotometry. FW, fresh weight. Data are expressed as the mean ± SD as determined from three independent biological replicates. Values in **(B,C)** that are significantly different between treatments and among lines within each treatment are marked with ^∗∗^*P* < 0.01 and ^∗^*P* < 0.05.

It is well documented that salt stress usually results in the excessive accumulation of ROS, which have deleterious effects on plant cells (Ashraf and Harris, 2004; Torun, 2018). Thanks to their antioxidant capacity, flavonoids play an important role against oxidative injury caused by salt stress (Dixon and Paiva, 1995). To determine the ROS scavenging capability of rutin, seedlings of WT plants with or without 200 mM NaCl treatment were stained with DAB or NBT. The histochemical staining suggested that salt stress dramatically increased the H_2_O_2_ or ${\text{O}}_{2}^{-}$ content compared to control. Conversely, supplementation with 100 μmol exogenous rutin during salt stress appeared to markedly decrease the levels of ROS (Figure 4A). The content of H_2_O_2_ or ${\text{O}}_{2}^{-}$ was measured using visible spectrophotometry. *NtMYB4* overexpressing transgenic plants showed a higher level of ROS (H_2_O_2_ or ${\text{O}}_{2}^{-}$ content) than WT plants under salt stress, while exogenous rutin effectively scavenged the ROS (Figure 4B,C). These results showed that rutin plays important roles in scavenging ROS.

### NtMYB4 Negatively Regulates the *NtCHS1* Activity

**FIGURE 5:**
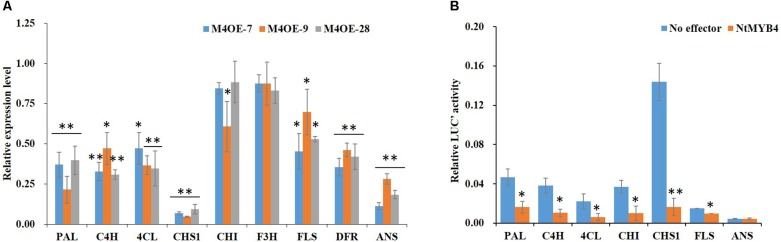
NtMYB4 negatively regulates the expression of flavonoid pathway genes. **(A)** The expression level of structural genes in *NtMYB4* overexpressing transgenic plants were significant inhibited compared to WT. The relative expression of structural genes for the WT control were set to 1. **(B)** Dual luciferase (LUC) assays of NtMYB4 (effector) and the promoters fused to pGreen luciferase plasmid (reporter). Empty vector was used as the effector in the control assay. The promoters of *PAL*, *C4H*, *4CL*, *CHI*, *CHS1*, *FLS*, and *ANS* genes were used in dual luciferase assays (*n* = 3). Data are expressed as the mean ± SD as determined from three independent biological replicates. Asterisks indicate that the value is significantly different from that of the control (^∗^*P* < 0.05, ^∗∗^*P* < 0.01).

In order to reveal the molecular mechanism mediated by NtMYB4 in regulating flavonoid biosynthesis, we determined the expression levels of flavonoid pathway genes and flavonoid accumulation in WT and *NtMYB4* overexpressing transgenic lines. qRT-PCR result showed that transcript levels of *PAL*, *C4H*, *4CL*, *CHS1*, *FLS*, *DFR*, and *ANS* were reduced in *NtMYB4* overexpressing transgenic plants. Notably, *CHS1* and *ANS* expression was significantly reduced by 94 and 89%, respectively (Figure 5A). Dual-luciferase assay was used to demonstrate whether or not NtMYB4 represses the promoter activity of *PAL*, *C4H*, *4CL*, *CHS1*, *CHI*, *FLS*, and *ANS* genes. Results showed that NtMYB4 significantly reduced the luciferase signal controlled by each promoter, except for that of the *ANS* gene. In particular, the luciferase signal controlled by the promoter of *NtCHS1* were reduced by 88.66% (Figure 5B). These results indicated that NtMYB4 regulates negatively the transcription of NtCHS1 and thus represses the flavonoid pathway.

### *NtCHS1* RNAi Silenced Transgenic Plants Showed a Similar Phenotype to *NtMYB4* Overexpressing Transgenic Plants Under Salt Stress

**FIGURE 6:**
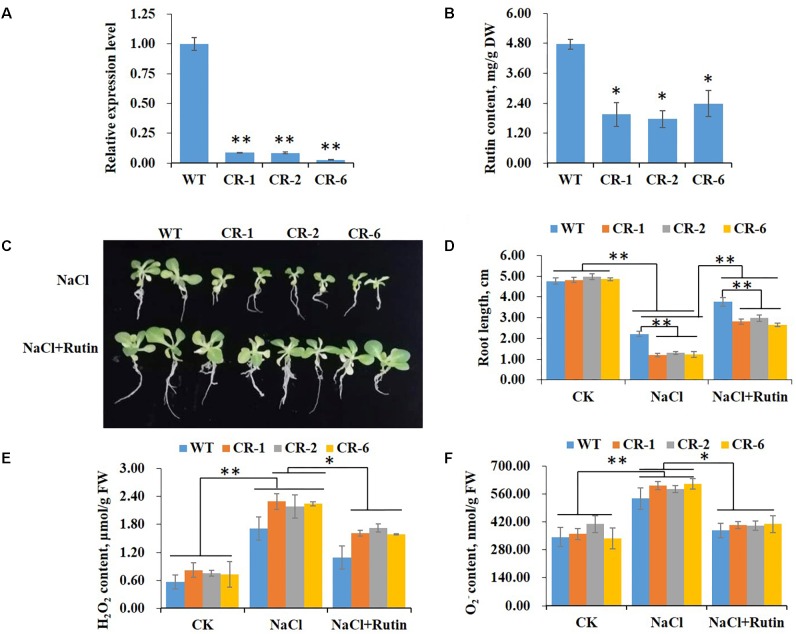
*NtCHS1* RNAi silenced transgenic plants show a similar phenotype to *NtMYB4* overexpressing transgenic plants in salt stress. **(A)** The expression level of *NtCHS1* decreased in RNAi silenced T_0_ transgenic tobacco leaves compared to WT. Asterisks in **(A)** indicate remarkable difference (^∗∗^*P* < 0.01) between *NtCHS1* RNAi transgenic lines and WT. **(B)** Rutin content decreased in *NtCHS1* RNAi silenced transgenic tobacco lines compared to WT. DW, dry weight. Asterisks in **(B)** indicate remarkable difference (^∗^*P* < 0.05) between *NtCHS1* RNAi transgenic lines and WT. **(C,D)** Root length of WT and *NtCHS1* RNAi transgenic plants treated with salt stress with or without exogenous rutin. Values in **(D)** that are significantly different between treatments and among lines within each treatment are marked with ^∗∗^*P* < 0.01 and ^∗^*P* < 0.05. **(E,F)** H_2_O_2_ or ${\text{O}}_{2}^{-}$ content of WT plants and *NtCHS1* RNAi silenced transgenic lines under control (CK) and salt treatment (NaCl) with or without rutin supplementation, measured by visible spectrophotometry. FW, fresh weight. Data are expressed as the mean ± SD as determined from three independent biological replicates. Values in **(E,F)** that are significantly different between treatments and among lines within each treatment are marked with ^∗∗^*P* < 0.01 and ^∗^*P* < 0.05.

Our previous study revealed that the expression level of *NtCHS1* was significantly induced by 200 mM NaCl treatment (Chen et al., 2017). To validate the function of *NtCHS1* in salt stress tolerance, we silenced the gene by RNA interference. The relative expression level of *NtCHS1* in the leaves of silenced transgenic tobacco lines was then analyzed using qRT-PCR. Three RNAi silenced lines (CR-1, CR-2, CR-6), out of the five independent *NtCHS1*-RNAi lines produced, were selected for further analyses, as they showed a significant decrease in the expression level of *NtCHS1* compared to WT plants (Figure 6A).

As shown in Supplementary Figure S3B, except for *PAL* and *DFR*, the expression of *4CL*, *CHI*, *F3H*, *FLS*, and *ANS* decreased significantly in *NtCHS1* RNAi lines. These lines also displayed a significant decrease in the levels of anthocyanins in the flowers and rutin in the leaves with respect to WT plants (Supplementary Figure S4B and Figure 6B). The results indicated that regulating the expression level of *NtCHS1* might alter the expression of major metabolic structural genes and the production of flavonoids in the rutin biosynthesis pathway.

To determine the role of *NtCHS1* in salt tolerance, seed germination rate and root length of WT and transgenic tobacco plants cultured on MS medium with or without the addition of 200 mM NaCl were measured. Severe suppression was observed in RNAi-silenced transgenic seeds, as the germination rate was decreased 95% under 200 mM NaCl. Moreover, the germination of *NtCHS1* RNAi-silenced transgenic seeds was delayed and it was almost abolished in the presence of 200 mM NaCl (Supplementary Figure S5). For root elongation, there was no obvious difference in seedling growth and root length between WT and transgenic lines under controlled condition (Figure 6D). In the 200 mM NaCl treatment, significant inhibition of root elongation was observed both in WT and RNAi transgenic lines. In addition, under the above condition RNAi transgenic lines displayed a significant reduction in root length with respect to the WT (Figure 6C,D). Supplementation with 100 μmol rutin in 200 mM NaCl MS medium enhanced root elongation of both WT and *NtCHS1* RNAi-silenced transgenic tobacco seedlings significantly (Figure 6C,D). Supplementation with rutin also reduced the ROS levels in the leaves of *NtCHS1* RNAi silenced transgenic plants (Figure 6E,F), as in *NtMYB4* overexpressing transgenic plants.

### *NtCHS1* Overexpressing Transgenic Plants Enhanced Salt Tolerance

**FIGURE 7:**
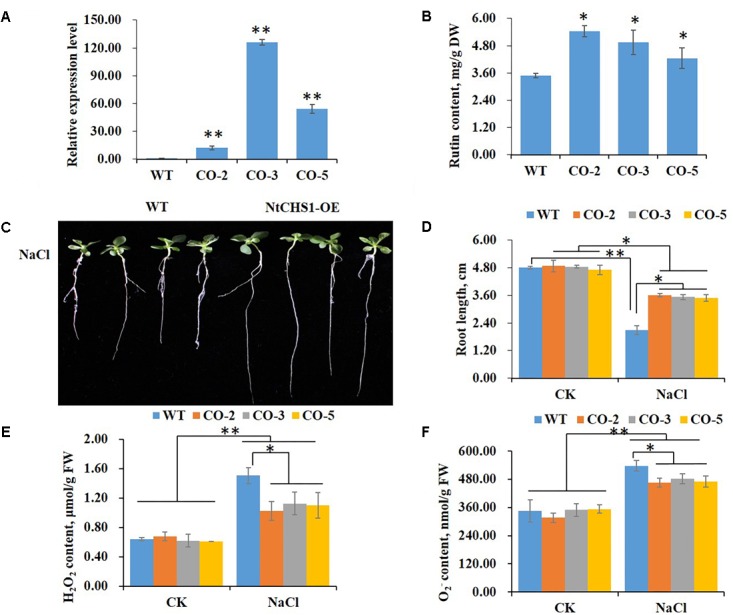
*NtCHS1* overexpressing transgenic plants significantly increase salt tolerance. **(A)** The expression level of *NtCHS1* in leaves was significantly increased in overexpressing T_0_ transgenic tobacco leaves compared to WT. Asterisks in **(A)** indicate remarkable difference (^∗∗^*P* < 0.01) between *NtCHS1* overexpressing transgenic lines and WT. **(B)** Rutin content increased in the leaves of typical *NtCHS1* overexpressing transgenic tobacco lines compared to WT. Asterisks in **(B)** indicate remarkable difference (^∗^*P* < 0.05) between *NtCHS1* overexpressing transgenic lines and WT. **(C,D)** Root length of WT and *NtCHS1* overexpressing transgenic plants (CO-3 line) treated with salt stress. Values in **(D)** that are significantly different between treatments and among lines within each treatment are marked with ^∗∗^*P* < 0.01 and ^∗^*P* < 0.05. **(E,F)** H_2_O_2_ or ${\text{O}}_{2}^{-}$ content of WT plants and *NtCHS1* overexpressing transgenic lines under control (CK) and salt treatment (NaCl), measured by visible spectrophotometry. FW, fresh weight. Data are expressed as the mean ± SD as determined from three independent biological replicates. Values in **(E,F)** that are significantly different between treatments and among lines within each treatment are marked with ^∗∗^*P* < 0.01 and ^∗^*P* < 0.05.

To further determine the function of *NtCHS1* in tolerance to high salt stress, we overexpressed the *NtCHS1* gene in tobacco. Three overexpression lines (CO-2, CO-3, and CO-5) were obtained. The results showed that the expression level of *NtCHS1* was significantly increased in the leaves of overexpressing lines compared to WT plants (Figure 7A). We also analyzed the expression levels of the structural genes involved in the flavonoid biosynthetic pathway in *NtCHS1* overexpressing lines. As shown in Supplementary Figure S3A, except for *PAL* with no obvious expression change, the expression of *4CL*, *CHI*, *F3H*, *DFR*, *FLS*, and *ANS* were significantly up-regulated in *NtCHS1* overexpressing lines. In addition, rutin content was significantly increased in overexpressing lines (Figure 7B). However, no obvious difference in anthocyanin content was detected between the three overexpression lines and WT (Supplementary Figure S4A).

The seed germination rate and root length of WT and *NtCHS1* overexpressing transgenic tobacco plants were also measured. These parameters did not change between WT and transgenic lines under controlled condition. Under 200 mM NaCl, the germination delayed with respect to the controlled condition in all plants, but the germination rate increased in *NtCHS1* overexpressing lines with respect to the control (Supplementary Figure S5). Notably, salt stress induced a significant inhibition of root elongation in both WT (55%), and, although less markedly (26%) in transgenic lines as well (Figure 7C,D). Furthermore, salt stress induced a significant increased in H_2_O_2_ or ${\text{O}}_{2}^{-}$ content in both WT and transgenic tobacco leaves. However, *NtCHS1* overexpressing transgenic lines had significant lower H_2_O_2_ or ${\text{O}}_{2}^{-}$ content than WT plants under salt stress (Figure 7E,F). These results showed that *NtCHS1* overexpression caused flavonoid accumulation and affected the rate of ROS scavenging.

## Discussion

In this study, we identified and characterized the *NtMYB4* gene from tobacco. Sequence analysis revealed that NtMYB4 contains the R2R3 domain, the zinc-finger domain as well as the EAR and LLsrGIDPxT/SHRxI/L motifs (Supplementary Figure S1), which are typical of sub-group 4 R2R3 MYB repressors (Jin et al., 2000; Zhou et al., 2015). 35S-UAS::GUS leaf transient assay and subcellular analysis showed that NtMYB4 is a nuclear localized transcription repressor in tobacco (Figure 1). Furthermore, dual-luciferase assay verified that NtMYB4 significantly decreased the promoting activity of most of the key genes in flavonoid biosynthesis, including *PAL*, *C4H*, *4CL*, *CHS*, *CHI*, and *FLS* (Figure 5B). qRT-PCR results confirmed that NtMYB4 overexpression significantly reduced the expression level of the six key genes (Figure 5A).

The expression of *CHS* was negatively regulated by subgroup 4 R2R3 TFs, including AtMYB4, AtMYB7, CsMYB4a, and FtMYB11 (Jin et al., 2000; Fornalé et al., 2014; Li M. et al., 2017; Zhou et al., 2017a). Our results indicated that NtMYB4 directly repressed *NtCHS1* gene expression to negatively regulate rutin biosynthesis in tobacco.

Transcription factors are initially vital in sensing salt and their expression levels are changed by salinity, leading to many tolerance responses (Deinlein et al., 2014). Little is known about how subgroup 4 R2R3 MYB TFs function in salt stress. The expression of *AtMYB7* was reported to be induced by salinity in *A. thaliana*, while *AtMYB4* expression was not induced (Fornalé et al., 2014; Kim et al., 2015). Here, the expression level of *NtMYB4* was found to be highly repressed by salinity (Figure 2A), indicating that NtMYB4 is a salinity-responsive TF. In order to demonstrate whether NtMYB4 functions in salinity tolerance, we overexpressed *NtMYB4* (Figure 2B) and investigated plant response to salinity. The results showed that *NtMYB4* overexpressing transgenic lines exhibited a much lower seed germination rate and shorter root length than WT plants (Figure 2C–F). In addition, the rutin content was significantly reduced in *NtMYB4* overexpressing transgenic lines. Supplementation with exogenous rutin in saline solution improved the salt tolerance of the *NtMYB4* overexpressing transgenic lines. These results indicated that inadequate content of rutin might directly result in the salt sensitivity of *NtMYB4* overexpressing transgenic lines.

It is well known that *CHS* expression is induced by various stimuli, including abiotic and biotic stress (Richard et al., 2000; Zabala et al., 2006; Dao et al., 2011; Chen et al., 2015). In our previous study, we found that *NtCHS1* was induced by high salt stress (Chen et al., 2017). Here, we further investigated the functions of *NtCHS1* transgenic plants under high salt stress, including overexpression or RNAi-silenced transgenic tobacco lines. Our data showed that *NtCHS1* RNAi-silenced transgenic tobacco lines, as *NtMYB4* overexpressing transgenic tobacco lines, exhibited reduced rutin contents and salt sensitivity (Figure 6). In addition, *NtCHS1* overexpressing transgenic tobacco lines exhibited increased rutin accumulation and higher salt tolerance than WT tobacco (Figure 7). These results further suggest that NtMYB4, by repressing *CHS*, decreases rutin biosynthesis and, in turn, salt tolerance.

Salinity leads to the overproduction of ROS in plants. ROS are highly reactive and toxic and result in oxidative stress (Gill and Tuteja, 2010). Evidence has accumulated that flavonoids are an important class of antioxidants, and have important roles in plant abiotic stress (Yang et al., 2001; Agati et al., 2012). AtMYB12 has been reported to confer salt and drought tolerance by increasing the levels of flavonoids in transgenic *A. thaliana* (Wang et al., 2016). Moreover, an extensive integrated analysis of single overexpression of AtMYB75 or AtMYB12, or double overexpression of AtMYB12 and AtMYB75, and tt4 as a flavonoid-deficient mutant, demonstrated that flavonoid over-accumulation was key to enhanced abiotic stress tolerance in Arabidopsis (Nakabayashi et al., 2014). The accumulation of flavonoids is enhanced by salinity (Colla et al., 2013), and the expression of the biosynthetic genes, including *PAL*, *CHS*, *CHI*, *DFR*, *FLS* and *ANS*, were shown to be increased in the present study (Supplementary Figure S3A). Similarly moderate salt stress increased rutin accumulation in buckwheat sprout (Lim et al., 2012), which indicated its function in plant salt tolerance. In the present study, higher ROS levels (H_2_O_2_ and ${\text{O}}_{2}^{-}$ activity) were detected in *NtMYB4* overexpressing (Figure 3A, 4) and *NtCHS1* RNAi-silenced transgenic tobacco, with reduced rutin content (Figure 6). Supplementation with exogenous rutin reduced H_2_O_2_ and ${\text{O}}_{2}^{-}$ activity in leaves. Moreover, *NtCHS1* overexpressing transgenic tobacco increased rutin accumulation and enhanced ${\text{O}}_{2}^{-}$ scavenging ability (Figure 7) under salinity. These results indicated that rutin exhibits strong ROS scavenging ability.

## Conclusion

We characterized *NtMYB4* functions as a repressor in salt responsiveness. The expression of *NtMYB4* was inhibited by salinity, resulting in the activation of *NtCHS1* transcription, followed by the accumulation of rutin to scavenge ROS. The results show that rutin is an important antioxidant and is effective in maintaining the balance of ROS in tobacco under salt stress. These results add more light to the roles played by flavonoids in the plant responses to saline stress.

## Author Contributions

SC and AY conceived and designed the research. SC, FW, YL, YQ, XP, CF, FL, and ZW performed the experiments and analyzed the data. SC and FL wrote the manuscript. YW, AY, and HL support for the research.

## Conflict of Interest Statement

The authors declare that the research was conducted in the absence of any commercial or financial relationships that could be construed as a potential conflict of interest.

## Footnotes

1 www.tobaccodb.org
