# Supplementary material for: NtMYB4 and NtCHS1 Are Critical Factors in the Regulation of Flavonoid Biosynthesis and Are Involved in Salinity Responsiveness
Source: Front Plant Sci. 2019 Feb 21;10:178. doi: 10.3389/fpls.2019.00178 (PMC6393349; doi:10.3389/fpls.2019.00178)
Supplement: Supplementary file 1 [file Presentation_1.PPTX]

## Slide 1
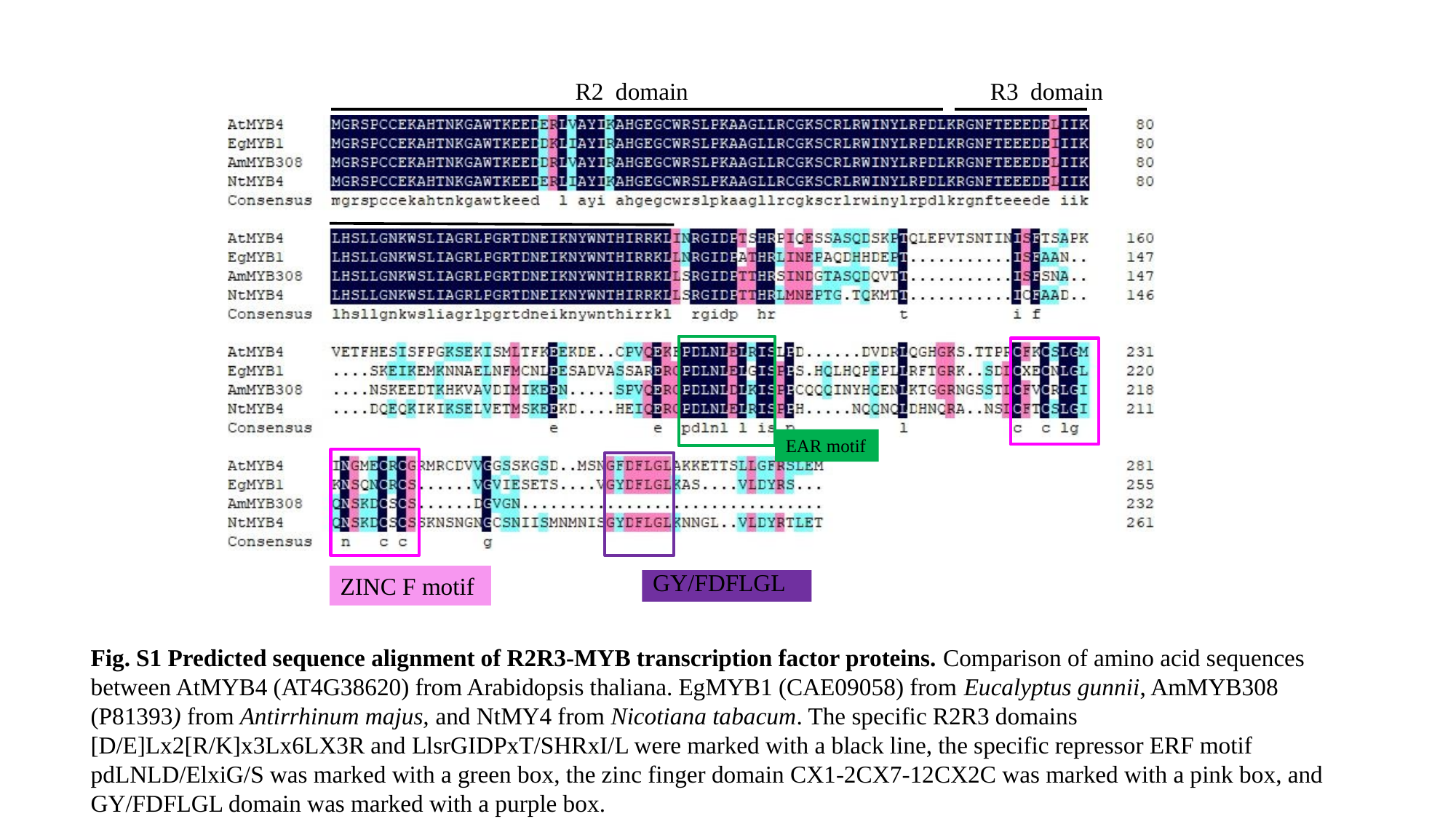

R2 domain
R3 domain
EAR motif
ZINC F motif
GY/FDFLGL
Fig. S1 Predicted sequence alignment of R2R3-MYB transcription factor proteins. Comparison of amino acid sequences between AtMYB4 (AT4G38620) from Arabidopsis thaliana. EgMYB1 (CAE09058) from Eucalyptus gunnii, AmMYB308 (P81393) from Antirrhinum majus, and NtMY4 from Nicotiana tabacum. The specific R2R3 domains [D/E]Lx2[R/K]x3Lx6LX3R and LlsrGIDPxT/SHRxI/L were marked with a black line, the specific repressor ERF motif pdLNLD/ElxiG/S was marked with a green box, the zinc finger domain CX1-2CX7-12CX2C was marked with a pink box, and GY/FDFLGL domain was marked with a purple box.

## Slide 2
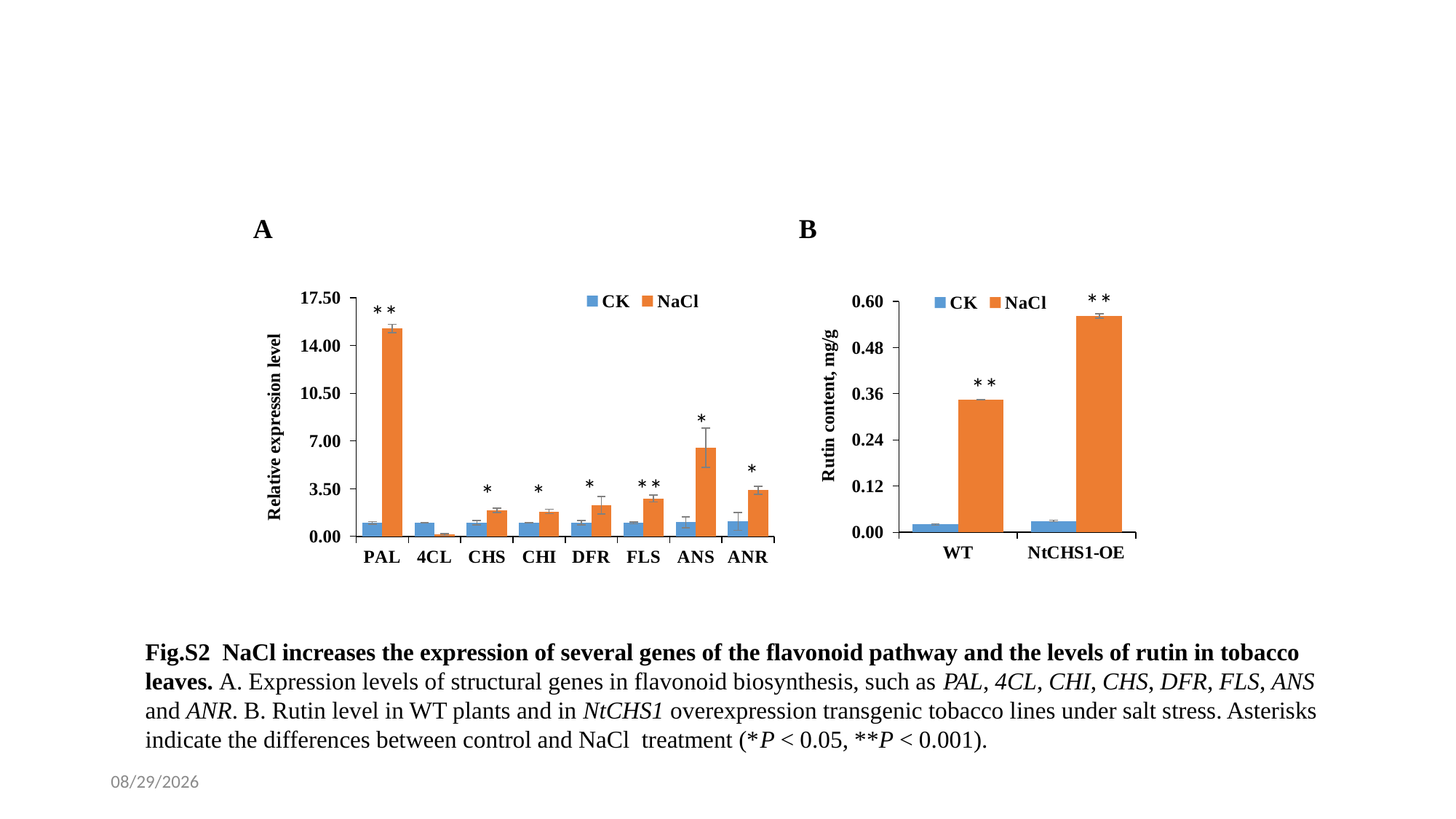

A
### Chart
| Category | | |
|---|---|---|
| PAL | 1.0018 | 15.2371 |
| 4CL | 1.0 | 0.1369 |
| CHS | 1.0088 | 1.9129 |
| CHI | 1.0002 | 1.8337 |
| DFR | 1.0103 | 2.2856 |
| FLS | 1.0012 | 2.7823 |
| ANS | 1.0402 | 6.507 |
| ANR | 1.1004 | 3.3846 |B
### Chart
| Category | | |
|---|---|---|
| WT | 0.0201 | 0.3444 |
| NtCHS1-OE | 0.0292 | 0.5621 |**
**
**
*
*
*
**
*
*
Fig.S2 NaCl increases the expression of several genes of the flavonoid pathway and the levels of rutin in tobacco leaves. A. Expression levels of structural genes in flavonoid biosynthesis, such as PAL, 4CL, CHI, CHS, DFR, FLS, ANS and ANR. B. Rutin level in WT plants and in NtCHS1 overexpression transgenic tobacco lines under salt stress. Asterisks indicate the differences between control and NaCl treatment (*P < 0.05, **P < 0.001).
2019/2/2

## Slide 3
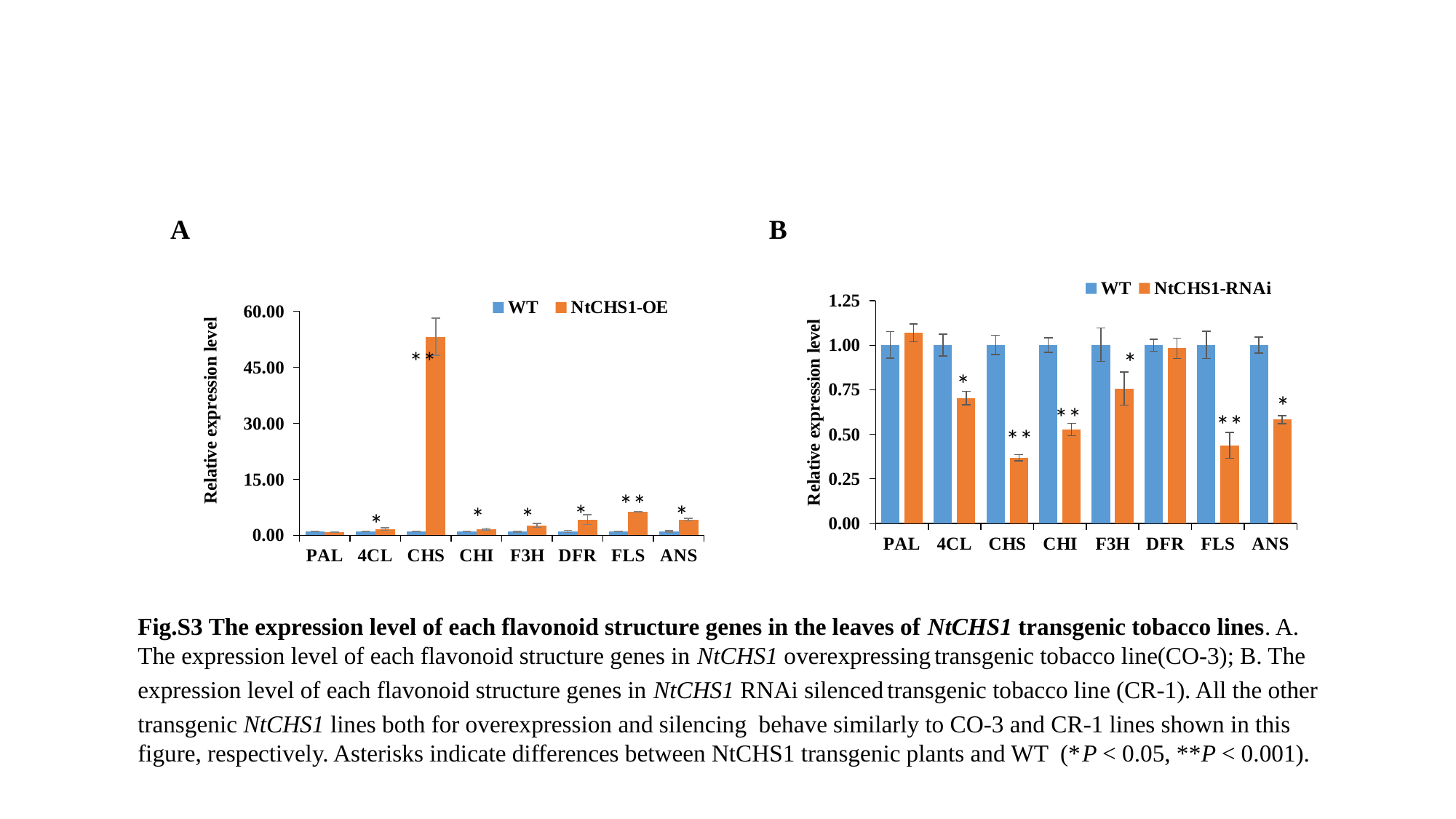

A
B
### Chart
| Category | | |
|---|---|---|
| PAL | 1.0018 | 1.0689 |
| 4CL | 1.0012 | 0.7042 |
| CHS | 1.001 | 0.3692 |
| CHI | 1.0006 | 0.5274 |
| F3H | 1.0028 | 0.7572 |
| DFR | 1.0003 | 0.9828 |
| FLS | 1.0021 | 0.4384 |
| ANS | 1.0007 | 0.5824 |
### Chart
| Category | | |
|---|---|---|
| PAL | 1.0009 | 0.8629 |
| 4CL | 1.0009 | 1.7095 |
| CHS | 1.0023 | 53.2333 |
| CHI | 1.0002 | 1.6591 |
| F3H | 1.0082 | 2.6265 |
| DFR | 1.0288 | 4.202 |
| FLS | 1.0002 | 6.3978 |
| ANS | 1.015 | 4.1999 |**
*
*
*
**
**
**
**
*
*
*
*
*
Fig.S3 The expression level of each flavonoid structure genes in the leaves of NtCHS1 transgenic tobacco lines. A. The expression level of each flavonoid structure genes in NtCHS1 overexpressing transgenic tobacco line(CO-3); B. The expression level of each flavonoid structure genes in NtCHS1 RNAi silenced transgenic tobacco line (CR-1). All the other transgenic NtCHS1 lines both for overexpression and silencing  behave similarly to CO-3 and CR-1 lines shown in this figure, respectively. Asterisks indicate differences between NtCHS1 transgenic plants and WT (*P < 0.05, **P < 0.001).

## Slide 4
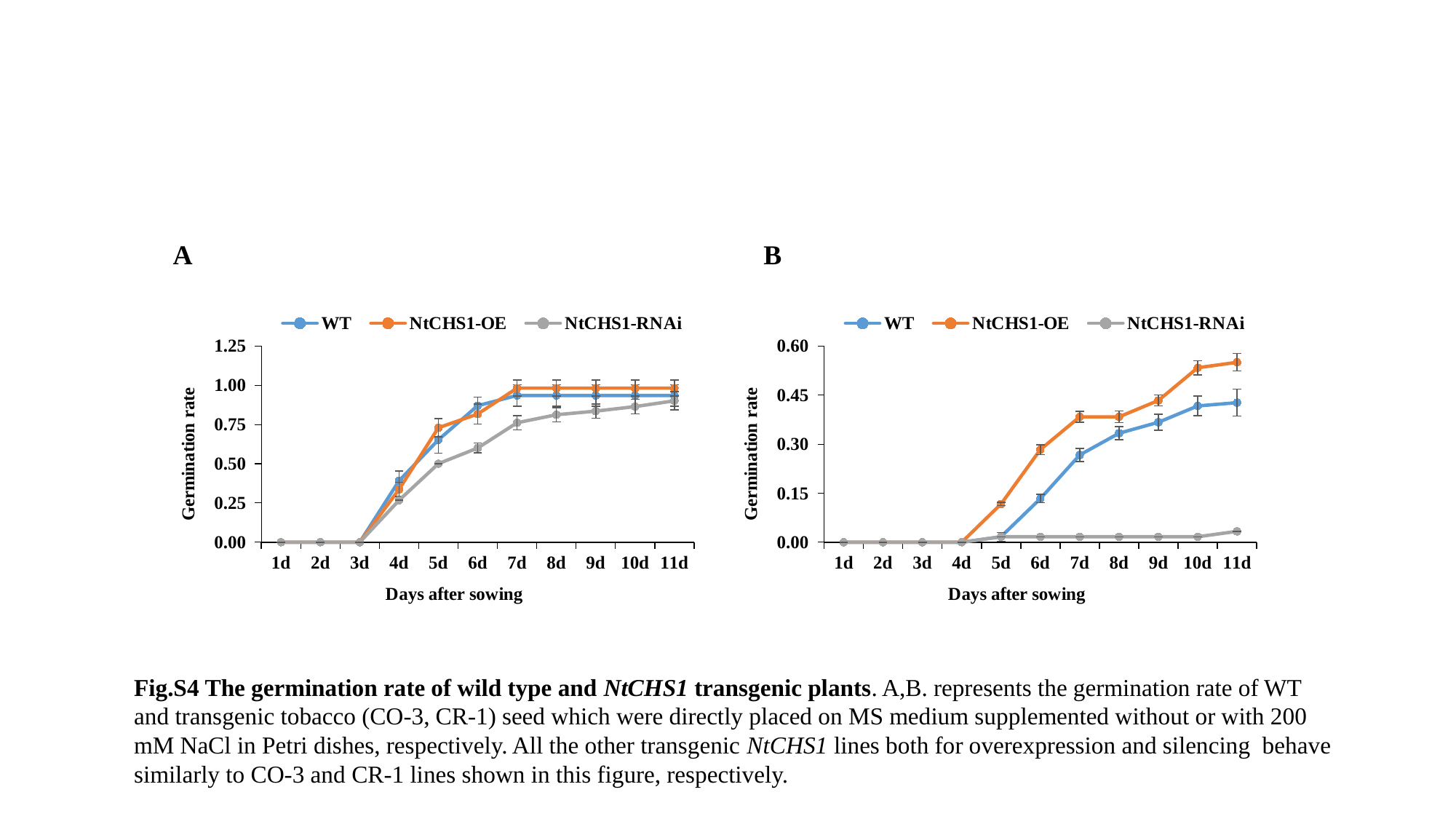

A
B
### Chart
| Category | | | |
|---|---|---|---|
| 1d | 0.0 | 0.0 | 0.0 |
| 2d | 0.0 | 0.0 | 0.0 |
| 3d | 0.0 | 0.0 | 0.0 |
| 4d | 0.3913 | 0.3364 | 0.267 |
| 5d | 0.6522 | 0.729 | 0.5 |
| 6d | 0.8696 | 0.8167 | 0.6 |
| 7d | 0.9348 | 0.9813 | 0.761 |
| 8d | 0.9348 | 0.9813 | 0.812 |
| 9d | 0.9348 | 0.9813 | 0.835 |
| 10d | 0.9348 | 0.9813 | 0.864 |
| 11d | 0.9348 | 0.9813 | 0.9005 |
### Chart
| Category | | | |
|---|---|---|---|
| 1d | 0.0 | 0.0 | 0.0 |
| 2d | 0.0 | 0.0 | 0.0 |
| 3d | 0.0 | 0.0 | 0.0 |
| 4d | 0.0 | 0.0 | 0.0 |
| 5d | 0.0167 | 0.1167 | 0.0167 |
| 6d | 0.1333 | 0.2833 | 0.0167 |
| 7d | 0.2667 | 0.3833 | 0.0167 |
| 8d | 0.3333 | 0.3833 | 0.0167 |
| 9d | 0.3667 | 0.4333 | 0.0167 |
| 10d | 0.4167 | 0.5333 | 0.0167 |
| 11d | 0.4267 | 0.55 | 0.0333 |Fig.S4 The germination rate of wild type and NtCHS1 transgenic plants. A,B. represents the germination rate of WT and transgenic tobacco (CO-3, CR-1) seed which were directly placed on MS medium supplemented without or with 200 mM NaCl in Petri dishes, respectively. All the other transgenic NtCHS1 lines both for overexpression and silencing  behave similarly to CO-3 and CR-1 lines shown in this figure, respectively.

## Slide 5
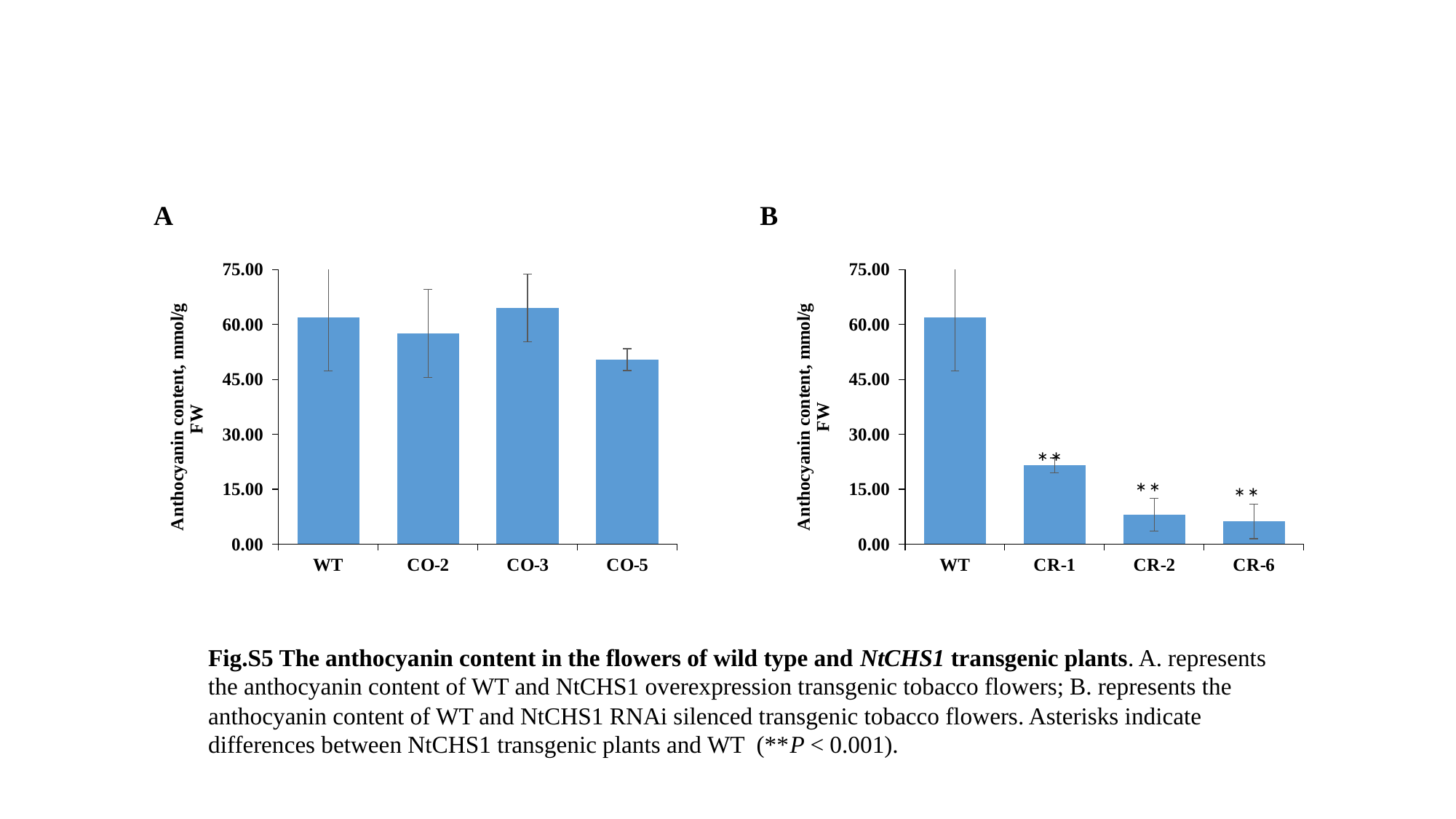

A
### Chart
| Category | |
|---|---|
| WT | 61.9621281795934 |
| CO-2 | 57.5588341379912 |
| CO-3 | 64.53142047172777 |
| CO-5 | 50.34236633163195 |B
### Chart
| Category | |
|---|---|
| WT | 61.9621281795934 |
| CR-1 | 21.543609222704532 |
| CR-2 | 8.0129372316196 |
| CR-6 | 6.2160458611827 |**
**
**
Fig.S5 The anthocyanin content in the flowers of wild type and NtCHS1 transgenic plants. A. represents the anthocyanin content of WT and NtCHS1 overexpression transgenic tobacco flowers; B. represents the anthocyanin content of WT and NtCHS1 RNAi silenced transgenic tobacco flowers. Asterisks indicate differences between NtCHS1 transgenic plants and WT (**P < 0.001).
