# Supplementary material for: NtMYB4 and NtCHS1 Are Critical Factors in the Regulation of Flavonoid Biosynthesis and Are Involved in Salinity Responsiveness
Source: Front Plant Sci. 2019 Feb 21;10:178. doi: 10.3389/fpls.2019.00178 (PMC6393349; doi:10.3389/fpls.2019.00178)
Supplement: Supplementary file 2 [file Table_1.DOCX]

**Supplemental Table 1.** Primers used in this study.

| **Genes** | **Primers** |
| --- | --- |
| NtMYB4-F | ATGGGAAGGTCACCATGTTGTG |
| NtMYB4-R | CTTAGTTTCCAATGTTCTATAG |
| NtMYB4OE-F | ATCTAGAATGGGAAGGTCACCATGTTGTG |
| NtMYB4OE-R | TGAGCTCCTTAGTTTCCAATGTTCTATAG |
| NtCHS1OE-F | ATCTAGAATGGTGACCGTCGAGGAATTTC |
| NtCHS1OE-R | TGAGCTCCTAAGTAGCAACACTGTGGAG |
| NtCHS1RNAi-F | GGGGACAAGTTTGTACAAAAAAGCAGGCTCGTATCACTAATAGCGAGC |
| NtCHS1RNAi-R | GGGGACCACTTTGTACAAGAAAGCTGGGTCGGGCATGTCTACACCAC |
| NtMYB4BD-F | CCGAATTCCCGGGGATCATGGGAAGGTCACCATGTTGTG |
| NtMYB4BD-R | CGCTGCAGGTCGACGGATCTTAGTTTCCAATGTTCTATAG |
| GAL4BD-F | CTCGACTCTAGAGGATCCATGAAGCTACTGTCTTCTATCG |
| GAL4BD-R | ATCGGGGAAATTCGAGCTCGATCCCCGGGAATTCGG |
| pMDC32-F | GACGCACAATCCCACTATCC |
| PMDC32-NtMYB4-R | ATCGGGGAAATTCGAGCTCTCACTTAGTTTCCAATG |
| NtMYB4-YFP-F | ATCTCGAGCTCAAGCTTCGAAATGGGAAGGTCACCATGTTGTG |
| NtMYB4-YFP-R | GTACCGTCGACTGCAGAATTCCTTAGTTTCCAATGTTCTATAG |
| pPAL-F | TTCCTGCAGCCCGGGGGATCCATTGAGGTCATCCGTTCTGC |
| pPAL-R | GGCCGCTCTAGAACTAGTGGACCGTGTAACGCCTTGTTTC |
| pC4H-F | TTCCTGCAGCCCGGGGGATCCAGAAGTCCTCGGAAATCA |
| pC4H-R | GGCCGCTCTAGAACTAGTGGGATAGCAAAGAATAACCCTA |
| p4CL-F | TTCCTGCAGCCCGGGGGATCCCTGGCTAAACTTACACTAC |
| p4CL-R | GGCCGCTCTAGAACTAGTGGCAGGGACGAGAACTGAAC |
| pCHS1-F | TTCCTGCAGCCCGGGGGATCCCATTTTTAAAAAGTTCATTATACA |
| pCHS1-R | GGCCGCTCTAGAACTAGTGGTTTCGCCGGAAAAAATGATGGAC |
| pCHI-F | TTCCTGCAGCCCGGGGGATCCTTCTCGGCTTGTTGCTTG |
| pCHI-R | GGCCGCTCTAGAACTAGTGGAACTTCACAAACTTCCCT |
| pFLS-F | TTCCTGCAGCCCGGGGGATCCCATAGCTCACTGGGTGGT |
| pFLS-R | GGCCGCTCTAGAACTAGTGGCTTTCAGTCCGCCACAGC |
| pANS-F | TTCCTGCAGCCCGGGGGATCCAGAGGAGAAGGTGCTACG |
| pANS-R | GGCCGCTCTAGAACTAGTGGTACCACCAGAGCAACACT |
| qNtMYB4-F | GGCTAATGAACGAACCTACTG |
| qNtMYB4-R | AGATTCAAATCAGGACACCGT |
| qNtPAL-F | ATTGAGGTCATCCGTTCTGC |
| qNtPAL-R | ACCGTGTAACGCCTTGTTTC |
| qNtC4H-F | GTGTTGCATTTGTGGTGGTTG |
| qNtC4H-R | TTCCGCTTAAGTTTACATCAG |
| qNt4CL-F | TCATTGACGAGGATGACGAG |
| qNt4CL-R | TGGGATGGTTGAGAAGAAGG |
| qNtCHS1-F | TTGTTCGAGCTTGTCTCTGC |
| qNtCHS1-R | AGCCCAGGAACATCTTTGAG |
| qNtCHI-F | GTCAGGCCATTGAAAAGCTC |
| qNtCHI-R | CTAATCGTCAATGCCCCAAC |
| qNtF3H-F | CAAGGCATGTGTGGATATGG |
| qNtF3H-R | TGTGTCGTTTCAGTCCAAGG |
| qNtDFR-F | AACCAACAGTCAGGGGAATG |
| qNtDFR-R | TTGGACATCGACAGTTCCAG |
| qNtFLS-F | GAACTTGAAGGGAAAAGGGG |
| qNtFLS-R | TCCCTGTAGGAGGGAGGATT |
| qNtANS-F | TGGCGTTGAAGCTCATACTG |
| qNtANS-R | GGAATTAGGCACACACTTTGC |
| Tob103-F | ATGAGAGAGTGCATATCGAT |
| Tob103-R | TTCACTGAAGAAGGTGTTGAA |
